# Investigation of a Medieval Pilgrim Burial Excavated from the *Leprosarium* of St Mary Magdalen Winchester, UK

**DOI:** 10.1371/journal.pntd.0005186

**Published:** 2017-01-26

**Authors:** Simon Roffey, Katie Tucker, Kori Filipek-Ogden, Janet Montgomery, Jamie Cameron, Tamsin O’Connell, Jane Evans, Phil Marter, G. Michael Taylor

**Affiliations:** 1 Department of Archaeology, University of Winchester, Winchester, United Kingdom; 2 Department of Archaeology, Durham University, Durham United Kingdom; 3 Research Laboratory for Archaeology and the History of Art, University of Oxford, Dyson Perrins Building, Oxford, United Kingdom; 4 McDonald Institute for Archaeological Research, University of Cambridge, Downing Street, Cambridge, United Kingdom; 5 NERC Isotope Geosciences Laboratory, Keyworth, Notts, United Kingdom; 6 Department of Microbial and Cellular Sciences, Faculty of Health and Medical Sciences, AX Building, University of Surrey, Guildford, Surrey, United Kingdom; Fondation Raoul Follereau, FRANCE

## Abstract

We have examined the remains of a Pilgrim burial from St Mary Magdalen, Winchester. The individual was a young adult male, aged around 18–25 years at the time of death. Radiocarbon dating showed the remains dated to the late 11^th^–early 12^th^ centuries, a time when pilgrimages were at their height in Europe. Several lines of evidence in connection with the burial suggested this was an individual of some means and prestige. Although buried within the *leprosarium* cemetery, the skeleton showed only minimal skeletal evidence for leprosy, which was confined to the bones of the feet and legs. Nonetheless, molecular testing of several skeletal elements, including uninvolved bones all showed robust evidence of DNA from *Mycobacterium leprae*, consistent with the lepromatous or multibacillary form of the disease. We infer that in life, this individual almost certainly suffered with multiple soft tissue lesions. Genotyping of the *M*.*leprae* strain showed this belonged to the 2F lineage, today associated with cases from South-Central and Western Asia. During osteological examination it was noted that the cranium and facial features displayed atypical morphology for northern European populations. Subsequently, geochemical isotopic analyses carried out on tooth enamel indicated that this individual was indeed not local to the Winchester region, although it was not possible to be more specific about their geographic origin.

## Author Summary

This multidisciplinary research article, involving biomolecular analysis, osteology, strontium and oxygen isotopic analyses and archaeology, examines the remains of a Pilgrim burial excavated from the medieval leprosy hospital of St Mary Magdalen, Winchester, UK. Radiocarbon dating showed the remains dated to the late 11^th^–early 12^th^ centuries, a time when pilgrimages were at their height in Europe. The *leprosarium* at Winchester is one of the earliest excavated examples from Western Europe and has been the subject of a series of recent academic papers. The site is remarkable for the high number of burials displaying skeletal lesions characteristic of leprosy (86%) and the state of preservation of biomolecular markers of the disease, including mycolipids and DNA. Genotyping of the *M*.*leprae* strain showed this belonged to the 2F lineage, today associated with cases from South-Central and Western Asia. Several aspects of the burial and dietary isotope analysis indicated this was an individual of some prestige and means; an unusual cranial morphology pointed to possible origin outside of the British Isles. Strontium and oxygen isotopic analyses confirmed he was not local to the Winchester area but were not able to pinpoint his precise origins. Overall these findings confirm the benefits of a multidisciplinary approach which allows investigation of the wider relationship between leprosy, medieval pilgrimage and *M*.*leprae* transmission.

## Introduction

We have recently examined cases of lepromatous leprosy (LL) recovered during excavations at the site of the St Mary Magdalen *leprosarium*, located to the east of Winchester, UK. Lepromatous or multibacillary leprosy is the more severe form of the disease, which lies at the opposite end of the spectrum from tuberculoid, or paucibacillary, leprosy. The two forms are manifestations of the same disease process, which is solely dependent on the immune response of the affected individual [1]. This leprosy hospital was an early foundation, probably dating back to the decades immediately following the Norman Conquest of 1066 [2]. The site is remarkable for the high number of burials displaying skeletal lesions characteristic of leprosy (86%) [3] and the state of preservation of biomolecular markers of the disease, including mycolipids and DNA. This has previously allowed detailed genotyping by conventional PCR [4] and for next generation sequencing (NGS) to be successfully applied to four of the more robust cases. This revealed a remarkably high level of genomic conservation in the leprosy bacillus over the last one thousand years [5,6].

**Fig 1:**
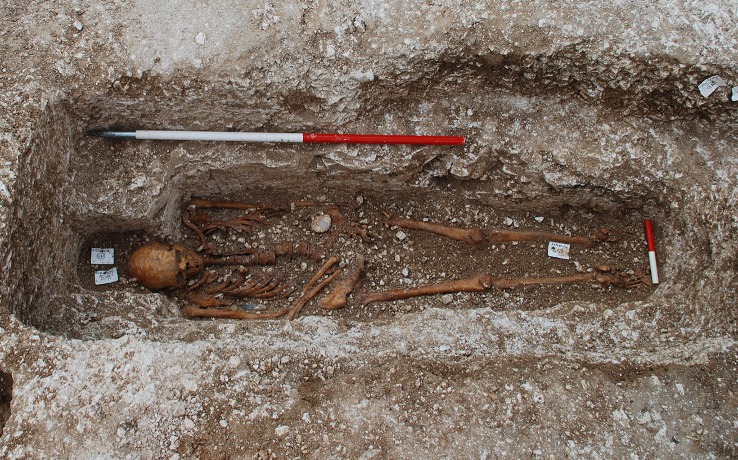
The burial of Sk27 *in situ*, showing the associated scallop shell (Credits: Magdalen Hill Archaeological Research Project/ MHARP).

The inhumation that is the subject of the current study, designated Sk27, had been buried in a chalk-cut anthropomorphic grave, similar to other individuals in the cemetery. This individual was interred together with a scallop shell, the traditional, and otherwise well-documented, symbol of a pilgrim who has made the journey to the shrine of St James in Santiago de Compostela, Spain (Fig 1). Pilgrimages to religious sites in medieval Europe were at their peak in the 12^th^ century. This, together with stratigraphic data for the burial and radiocarbon dating, discussed later, suggested a time of death sometime in the first half of the 12th century.

In the current study, we have looked in depth at the strain of leprosy causing disease in this pilgrim burial and have used radiocarbon dating and dietary isotopes to better relate these observations to the phylogeny of *M*.*leprae* and likely origins of this individual. As the skeletal lesions were minor, we have also sought evidence for other pathogens which may have contributed towards the early death of the individual. The findings are compared to other cases recently studied from this site. Together, these results add to our understanding of isolates behind the widespread nature of European leprosy in the high Middle Ages and in particular of a rare lineage which is less common amongst extant strains. The study concludes by considering these findings in their wider historical and comparative context

## Methods

### Osteology

All necessary permits were obtained for the field studies, including a license (-0070) to exhume and retain human remains, provided by the Ministry of Justice, 102 Petty France, London SW1H 9AJ. The site of St. Mary Magdalen, Winchester is designated by the site code AY352. The skeletal remains, artefacts, environmental samples and paper archive are held in a permanent repository in the Department of Archaeology, University of Winchester.

The skeleton that is the subject of this paper (designated AY352/11/14 (489) Sk27) was excavated by hand from a sealed context and was from a single, west-east aligned, chalk-cut grave with a head-niche and inner ledge, within the northern cemetery area of the site. The grave had largely truncated an earlier grave (Sk26), of which only the head-niche, part of the northern side of the cut and a humerus remained *in situ*. (The majority of Sk26 was recovered as disarticulated bone from the grave fill of Sk27). The grave of Sk27 had subsequently been partially covered by the construction of the north wall of the medieval chapel (ca. 1160s), leaving the west end of the grave under the doorway of the building. A number of other graves that predated the building of the chapel or other hospital buildings had been emptied out before walls or floors were constructed over them, so it is interesting to speculate as to whether Sk27 was deliberately left *in situ*. Recent excavations conducted at the eastern end of the medieval chapel, have revealed evidence for another structure, possibly an earlier phase of chapel dating to the original foundation in the late 11^th^ century. It is possible that Sk27 was associated with this structure.

The skeleton was in a supine and extended position, with the left upper limb at the side and the right upper limb flexed at the elbow with the hand on the pelvis. A scallop shell (*Pecten maximus*, a species with a distribution along the European Atlantic coast from northern Norway to the Iberian peninsula [7]), pierced with two holes, was found on the left side of the pelvis (see Fig 1). The individual was very well preserved and nearly complete, with only the maxillary left lateral incisor, two hand phalanges and three pedal phalanges being absent.

Two skeletons, Sk1 and Sk12 (selected as controls for the biomolecular aspects of this study), were excavated by hand from sub-rectangular chalk-cut graves within the chapel. Sk1 had been buried within a “shouldered” coffin (evidenced by the pattern of the coffin nails) within a grave that cut through and disturbed a number of earlier graves. This would suggest that the burial dated to the 17^th^ century, when the hospital/almshouse was in decline and was subject to reuse as a prisoner of war camp during the Anglo-Dutch wars of the latter half of that century [8]. The skeleton was supine and extended with the upper limbs straight at the sides and the hands on the pelvis. Sk12 was excavated from a plaster-lined grave covered with a Purbeck marble slab but was recovered as disarticulated bones, together with the disarticulated remains of a 2–3 year old child (Sk13) suggesting that the grave had been robbed at some point before the chapel was demolished in the late 18^th^ century.

As the site was known to be a *leprosarium* and the possibility of finding individuals with skeletal evidence of leprosy was anticipated prior to excavation, the graves were subject to extensive sampling of the grave fills, which were then floated and hand-sorted. This allowed for near-complete retrieval of the small bones of the hands and feet, which are invaluable for the correct diagnosis of leprosy, particularly in its earlier stages.

The skeletons underwent osteological examination in the Department of Archaeology, University of Winchester, Winchester, UK [3]. A detailed inventory of skeletal elements was completed using both written and diagrammatic *proforma*. Determination of sex in the adult individuals was undertaken using the methods of Phenice [9] for features of the pubic bone, Buikstra and Ubelaker [10] for the greater sciatic notch, and Acsádi and Nemeskéri [11] for features of the cranium and mandible. Individuals were then assigned one of five sex classifications: definite male (M); possible male (? M); indeterminate (?); possible female (? F); definite female (F).

Estimation of age in the adult individuals was undertaken using the methods of Brooks and Suchey [12] for the pubic symphysis and Lovejoy *et al*. [13] for the auricular surface. Estimation of age in the non-adult individuals was undertaken using long bone lengths, dental development and epiphyseal fusion, as outlined in Scheuer and Black [14]. Individuals were then placed into one of the following age categories, using the Museum of London Archaeology guidelines, advocated by Falys and Lewis for adults [15]: foetal (up to 36 weeks gestation); perinate (from 36 weeks gestation to 1 month); infant (1 month to 1 year); young child (1–5 years); older child (6–11 years); adolescent (12–17 years); young adult (18–25 years); young middle adult (26–35 years); old middle adult (36–45 years); mature adult (46+ years).

Metric data from the crania, mandibles and post-cranial skeletons were recorded using the lists given in Buikstra and Ubelaker [10], with additional measurements from the crania being recorded according to the criteria given in Wright [16]. The discriminant functions programs, FORDISC [17] and CRANID [16] were used to assist in the analysis of possible ancestral traits and geographic origins. In using this data, it is recognized that genetic admixture has resulted in considerable overlap between traits that are considered characteristic of major ancestral groups and therefore “pure” ancestral classifications are neither possible nor desired. There is no doubt that early craniometric studies were tainted by racism [18] but when modern methods and attitudes are used, it can still provide valuable additional information on the possible population affinity of archaeological individuals (see the publications produced as part of the recent Roman Diaspora Project for examples of modern studies using craniometric data in combination with other techniques to assess patterns of migration and diversity in archaeological populations [19, 20]. Stature was calculated using the methodology of Trotter [21]. All evidence for pathology and trauma was documented in detail through the use of descriptions and photographs and a variety of sources were used to diagnose the conditions represented. These sources are referenced at the appropriate places within the text. In particular, the individual was carefully examined for any skeletal manifestations of leprosy, including the rhino-maxillary syndrome and changes to the hands and feet (described in detail elsewhere [3,4]).

### Biomolecular study

#### Sampling

Sampling of the Pilgrim burial (Sk27) along with the two control burials, Sk1 and Sk12 took place in the Department of Archaeology, University of Winchester, Winchester, Hampshire, UK. To maximize the chances of recovering *M*. *leprae* pathogen DNA, bone samples were taken from around the rhino-maxillary area from all three individuals. Bone fragments were taken from the nasal conchae of Sk1 (110 mg), Sk12 (50 mg) and Sk27 (50 mg). Further samples (all 50mg) were taken from the foot, rib and skull from Sk27 to assess the likely extent of the disease. An additional 50 mg sample was also taken from the maxillary palatine process of Sk1.

Steps were taken from the outset to minimize the chances for cross-contamination between cases, during sampling and subsequently in the molecular biology laboratories. These have been documented previously [22] but a brief overview is given below.

#### Measures to prevent contamination

Precautions were taken from the outset, including the time of sampling. Disposable scalpels and sterile tubes were used to sample and store bone fragments respectively. Different laboratories and equipment were used for DNA extraction, PCR set-up and post PCR stages. These were physically removed from each other and had separate air supplies and removal. PCR assays were set up in either duplicate or triplicate and this included extraction blanks taken through the DNA preparation stages and non-template controls (water) to monitor for random contamination. Reactions were monitored on the real-time platform to ensure contamination-free blanks. Positive controls were not included.

#### DNA extraction

We used the Nuclisens extraction kit from bioMérieux to prepare DNA extracts from all bone samples [22]. The extracts were stored in LoBind Eppendorf tubes to minimize losses of DNA onto the tube walls. The samples were stored at -20C until assayed for pathogen DNA.

1. The multi-copy element RLEP was used to screen for evidence of *M*. *leprae* DNA. The method and primer sequences have been previously reported [22]. In the present study, the intercalating dye EVAGreen (Biotium Inc.) was used to monitor product formation, with dissociation curve analysis used to confirm melt temperatures of products. A second real time PCR method, for the single copy 18-kDa-antigen gene, was used to confirm presence of leprosy DNA and to assess suitability for further genotyping [4].

2. Evidence for *Mycobacterium tuberculosis* (MTB) complex was sought using real time PCR for the multi-copy element IS*1081*. This was performed as described [23] with modifications. These included the use of new forward (5’-ctgaagccgacgccctgtgc-3’) and reverse (5’-tggcggtagccgttgcgc-3’) primers to amplify extremely degraded DNA fragments (79 bp). A dual-labelled hybridization probe 5’-(HEX]attggaccgctcatcgctgcgttcgc[BHQ1)-3’ was used to follow product formation [24].

3. The extracts from Sk27 were also tested for a number of other pathogens by PCR and these are listed in Table 1.

**Table 1:** Summary of PCR methods used to screen for other pathogens.

Pathogen	Disease	Gene target	Reference	Amplicon size (bp)	Cycles	Reporter
*Brucella spp.*	Brucellosis	IS*711*	[105].	108	41	EVA Green / probe
*Treponema pallidum*	Treponematosis	15-KDa lipoptotein gene	[106].	120	41	EVA Green
*Burkholderia pseudomallei*	Melioidosis	Chromosome 2	[107].	81	41	EVA Green
*Leishmania spp.*	Leishmaniasis	Minicircle kinetoplast	[108].	116	41	EVA Green
*Falciparum spp.*	Malaria	18S rRNA gene	[109].	135–138	41	EVA Green
HBV	Hepatitis B	S gene	[110].	98	43	EVAGreen

PCR methods were performed on an Mx3005P real-time platform (Agilent Technologies, Wokingham, UK) in a final volume of 25μl [22]. Routine agarose gel electrophoresis (3%) was used to determine the sizes of PCR products.

#### Genotyping of Sk27

Both single nucleotide polymorphism (SNP) analysis and variable nucleotide tandem repeat typing (VNTR) was undertaken on the remains.

1. SNP typing. A series of polymorphic SNP loci were amplified and sequenced to determine the main SNP type and subtype. These loci and the primer sequences have been reported [4,25,26]. Specific PCR products were purified by agarose gel electrophoresis (3%) and Sanger sequenced with both forward and reverse primers by Beckman Coulter Genomics, Takely, Essex, UK.

2. VNTR typing. Two microsatellite and one minisatellite repeat loci were analyzed. These were: (i). (AGA)20, ML2344-ML2345. (ii). (GTA)9, ML2172-ML2173 and (iii). The ML0058c locus also known as 21–3 with a variable number of a 21 bp tandem repeat sequence (5’-tgatcaacttgattcctggct-3’). Primer sequences and PCR details have been previously published [22].

### Stable isotope analysis

#### Carbon and nitrogen isotopes

Carbon and nitrogen stable isotope analyses were conducted on bone collagen preserved in the remains of individual Sk27. Samples from 42 other humans excavated from the same site were also taken at the same time for a separate research project. For the purposes of this paper this material can be used for comparison with Sk27.

Small samples of approximately 500mg were taken from Sk27 and 42 other sets of human remains excavated from St Mary Magdalen, Winchester. Where possible, small, loose fragments of rib were used, however the exceptional preservation of some ribs necessitated the use of a handheld rotary saw to remove fragments of appropriate size. Pathological bone was avoided, as the effect of pathological alterations on isotope ratios is inadequately understood [27].

Collagen was extracted from the samples in accordance with the standard operating procedure of the Dorothy Garrod Laboratory for Isotopic Analysis at the McDonald Institute for Archaeological Research, University of Cambridge. The samples were first sandblasted using aluminum oxide abrasive powder to remove surface deposits, then dematerialized in 0.5M aq. hydrochloric acid 4°C. The acid was changed every 2–4 days until demineralization was complete, taking from 24 hours to 4 weeks depending on the sample. Samples were then gelatinized in 8ml pH 3.0 water at 75°C for 48 hours, the solution filtered off and lyophilized (freeze-dried).

Samples were isotopically analyzed in triplicate at the Godwin Laboratory, Department of Earth Sciences, University of Cambridge, using a Thermo Finnigan Delta V plus isotope ratio mass spectrometer, coupled in continuous flow mode to a Costech Elemental Analyzer. Replicate analyses of laboratory and international standards showed that analytical precision was <0.2‰ for both δ^13^C and δ^15^N.

#### Strontium and oxygen isotopes

The right mandibular third molar was removed from its alveolar socket by hand. The tooth was macroscopically analyzed for evidence of pathology and photographed in the occlusal, buccal, lingual and apical aspects before sample preparation.

Sections of core enamel were extracted in the Sample Preparation Laboratory in the Department of Archaeology, Durham University. There was no evidence of pathology on the tooth. Tooth sample preparation followed the guidelines of Montgomery [28]. Tooth surfaces were first cleaned using tungsten carbide burrs and then enamel was sectioned using diamond edged dental saws. All surface enamel and any adhering dentine were mechanically removed. After cleaning, samples were weighed to record yield (Table 2). Processed chips of core enamel were sealed in Eppendorf microtubes and transferred to the (class 100, HEPA-filtered) laboratory facilities at the NERC Isotope Geosciences Laboratory (NIGL) at British Geological Survey (Keyworth, Nottinghamshire, UK) for further preparation.

**Table 2:** Tooth formation and age-at-death based upon data from Al Qahtani et al. [85].

Skeleton	Tooth	Tooth Formation	Age-at-death, based upon tooth formation	Pathology	Sr sample weight (mg)	O sample weight (mg)
Sk27	Right Mandibular M3	A ½: Root apex closed with wide PDL^1^	22.5–23.5 years	Linear enamel hypoplasia, dental calculus	19.4 mg	12.5 mg

^1^PDL = periodontal ligament space.

For strontium isotope analyses, the sections of core enamel were then further prepared and measured according to Evans and co-workers [29]. In a clean laboratory, the enamel sample was first cleaned ultrasonically in high purity water to remove dust, rinsed twice, dried down in high purity acetone and then weighed into pre-cleaned Teflon beakers. A known amount of ^84^Sr tracer solution was added to each sample, which was dissolved in Teflon distilled 8 M HNO3. The sample was converted to chloride using Quartz distilled in 6M HCl and then taken up in 2.5 M HCl. The Sr was extracted using conventional ion exchange methods. The isotope composition and concentrations were determined by Thermal Ionization Mass spectroscopy (TIMS) using a Thermo Triton multi-collector mass spectrometer. The international standard for ^87^Sr/^86^Sr, NBS-987, gave a value of 0.710251 ± .000005 (n = 19, 2s) during the analysis of these samples. Procedural blank values were less than 100pg.

Approximately 1.5 milligrams of powdered enamel was loaded into a glass vial and sealed with septa. The vials were transferred to a hot block at 90°C on a GV Multiprep system. The vials were evacuated and 4 drops of anhydrous phosphoric acid added. The resultant CO_2_ was collected cryogenically for 14 minutes and transferred to a GV IsoPrime dual inlet mass spectrometer. The resultant isotope values are reported as ^18^O/^16^O‰ normalized to the PDB scale using a within-run calcite laboratory standard (KCM) calibrated against SRM19, NIST reference material. These ratios are converted to the SMOW scale using the published conversion equation of Coplen [30]: SMOW = (1.03091 x ™^18^O _VPDB_) +30.91. Analytical reproducibility for this run of laboratory standard calcite (KCM) is 0.09‰ (1 σ, n = 6) for ^18^O_SMOW_ and ± 0.05‰ (1s, n = 6) for ^13^C_PDB._ The reproducibility of the enamel, based on average 1SD of five duplicate pairs is ± 0.07‰. The carbonate oxygen results ^18^O_SMOW(c)_ are converted to phosphate values ™^18^O_SMOW (p)_ using the equation ™^18^O_SMOW (_p_)_ = 1.0322*™^18^O_SMOW (_c_)_− 9.6849 [31]. Combining the uncertainty of the carbonate analysis (± 0.07‰) with typical uncertainty for phosphate analysis (± 0.29‰ 1SD, value from reference [31]) produces an overall uncertainty of the derived phosphate values of ± 0. 3‰ (1SD). The phosphate values are used to calculate drinking water values using the equation of Daux and colleagues [32] (DW = 1.54*(™^18^O_SMOW(p)_ -33.72). The calculation of drinking water values involves considerable uncertainties [33] and the values should be used as guidance only.

## Results

### Osteology

Sk27 –The individual was found to be a young adult male (18–25 years) with a stature of 168.9 ± 2.99cm (calculated from the femur + tibia). This is within the expected range of average male stature for the period of 171cm, which was calculated by Roberts and Cox [34] from 8494 individuals from 34 British sites dating from c.1050-c.1550.

**Fig 2:**
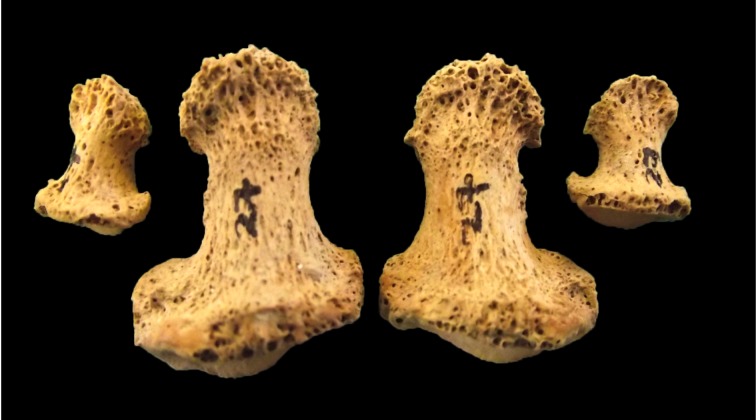
Sk27: Distal foot phalanges showing porosity and beginnings of some bone resorption (Credit: MHARP)

There were no lesions characteristic of leprosy in the rhino-maxillary area or hands. The lesions in the feet were restricted to the distal pedal phalanges, particularly those of the hallux, which were found to exhibit porotic changes and some resorption of the distal ends as a result of the initiation of achro-osteolysis. While such changes are not normally considered to be pathognomic for the disease, similar alterations are found in a number of other individuals from the hospital cemetery who do exhibit early-stage rhino-maxillary changes (also recorded by Ortner [35] in individuals from Chichester) and have been recorded as typical for early stage leprosy in the clinical literature (Fig 2;[36]). The distal shafts of the tibiae of the individual also demonstrated evidence for remodelled periosteal lesions, with similar lesions also being found on the distal shaft of the left femur and the proximal shaft of the left fibula. These lesions form in response to inflammation or infection and can have numerous aetiologies, including trauma [37], although it has also been suggested that, where the major focus of the reactive bone is on the distal ends of the bones, a diagnosis of leprosy should be considered [35]. If the lesions in this individual are to be ascribed to leprosy, it would suggest that the soft tissue manifestations of the disease, with associated inflammation and infection, were much more developed than the bony lesions.

**Fig 3:**
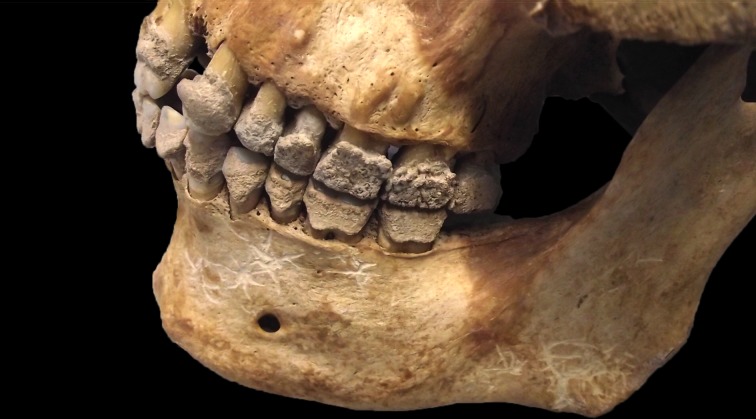
Sk27 skull showing heavy deposition of calculus on the left side of the mandibular and the maxillary dentition, particularly on the latter, where it exhibits a nodular appearance (Credit: MHARP)

There was evidence for a large amount of dental calculus on the left maxillary and mandibular dentition (Fig 3). On the buccal surfaces of the teeth this had a nodular appearance and there was also evidence for calculus within the pits and furrows of the occlusal surfaces. This indicates that the calculus had not been smoothed down by the action of the cheek in normal masticatory function and that the entire occlusal surfaces of the dentition had at one point been covered by calculus. The left mandibular third molar had been lost ante-mortem and the alveolar bone was in the process of remodelling. It is possible that this tooth had been affected by dental caries that caused an amount of pain to the individual and prevented them from using the left side of their mouth during eating. The loss of this tooth allowed normal masticatory function to be restored by the time of the individual’s death (as evidenced by the removal of calculus from the occlusal surfaces, leaving traces in the inaccessible pits and furrows).

However, high levels of dental calculus have also been recorded in archaeological individuals with skeletal evidence for leprosy, where it is thought to relate to a soft, pulpy hospital diet, poor dental hygiene as a result of the leprous involvement of the oral cavity (and also difficulty in using the hands to perform dental hygiene [38]), and mouth-breathing due to chronic inflammation of the nasal passages [3,39]. High levels of dental calculus are also found in modern individuals with the disease [38,40,41]. Dental calculus with a nodular appearance has been recorded in a number of other individuals from St Mary Magdalen with skeletal evidence for leprosy and was also seen in one individual with leprosy from St James and Mary Magdalene, Chichester, where it was argued to be related to paralysis of the facial muscles or loss of soft tissues of the cheek [39]. Therefore, it is possible that a similar aetiology may account for these changes in Sk27, also supporting the assertion that the soft tissue manifestations of leprosy may have been more substantial than the bony lesions would suggest.

The right maxillary central incisor showed resorption of the apical end of the root and the associated alveolar socket was shallow and porous in appearance. A number of individuals from St Mary Magdalen show evidence for constriction and abnormal development of the roots of the maxillary incisors and canines with associated shallow and porous alveolar sockets, which has been identified as *leprogenic odontodysplasia* [3,4]. In this case, however, the root does not show any evidence of constriction or abnormal development and the appearance is more consistent with resorption subsequent to trauma [42,43].

The individual had evidence for degenerative changes, in the form of porosity and osteophyte development, of the first and second cervical and the thoracic vertebrae, the coccyx, the acromial end of both claviculae and the anterior of the olecranon process of both ulnae. The mid-thoracic and lumbar vertebrae of the individual also had evidence for Schmorl’s nodes (depressions of the surfaces of the vertebral bodies).

There was evidence for entheseal changes, in the form of enthesophyte development, to the attachment sites for *M*. *triceps* (the muscle principally responsible for extension of the arm) on both ulnae, *M*. *quadriceps femoris* (responsible for extending the knee) on both patellae and for *tendo calcaneus* (responsible for plantar flexion of the foot and flexion of the knee) on both calcanei.

The attachment sites for *M*. *deltoideus* (involved in abduction, extension and rotation of the shoulder) on the right scapula and the left clavicle, for the costoclavicular ligament (responsible for stabilising the shoulder) on both claviculae, for the common extensors (responsible for extending the forearm) on the right humerus, for *M*. *vastus medialis* (involved in extending the knee) on the left femur, for *M*. *adductor magnus* (largely responsible for adduction and rotation of the thigh and involved in extension of the hip) on both femorae and for *M*. *abductor hallucis* (involved in abduction and flexion of the Hallux) and *M*. *flexor digitorum brevis* (responsible for flexion of the lateral four toes) on the left calcaneus, also showed an increase in robusticity and development when compared to other attachment sites.

The presence of degenerative changes in a young adult would seem to indicate an unusual degree of muscular and skeletal wear and tear related to increased levels of activity. That these changes do not simply reflect generalized wear and tear is supported by many studies demonstrating that an increase in the prevalence and severity of degenerative changes is strongly correlated with increasing age [44,45]. The assertion that these changes in Sk27 are related to activity patterns may also be supported by the finding that they are restricted to certain anatomical areas, namely the vertebral column, shoulders and elbows (e.g. see refs [46–48] for studies where specific patterns of degenerative changes are linked to specific activity patterns, although a number of other studies have disputed this link, [49–51]). The presence of Schmorl’s nodes, which have a complicated aetiology but are presumed to be related to compressive forces to the back, including bending and twisting while supporting a weight [52,53], may also support this interpretation, as would the presence of entheseal changes. These also have a complicated aetiology, with suggestions that they are more strongly correlated with age and sex than activity [54,55], although recent studies have also found that activity may indeed play a part in their development [56,57].

**Fig 4:**
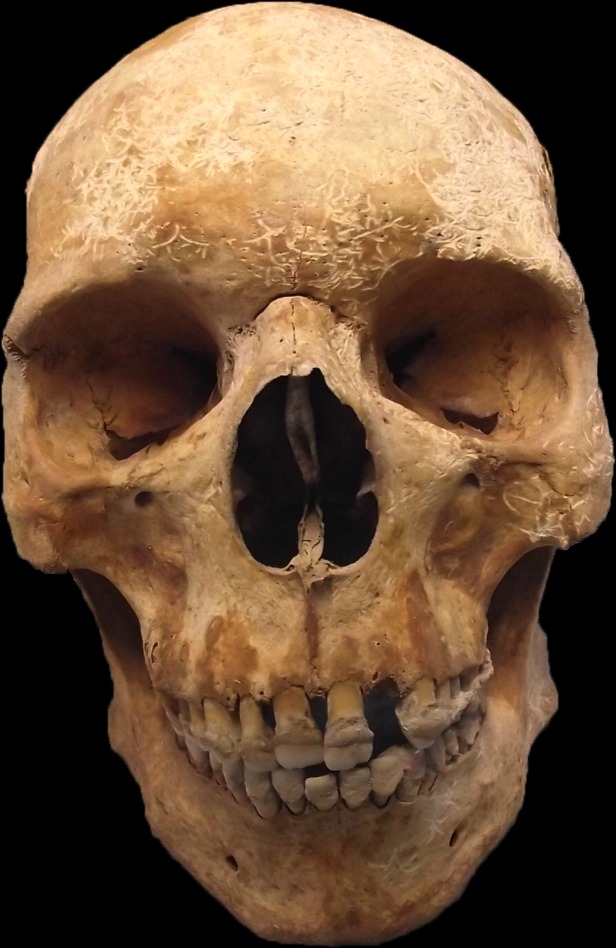
Frontal view of Sk27 skull, showing preservation of the anterior nasal spine and no obvious signs of *facies leprosa*. The appearance of the cranial morphology was notably different to other individuals in the same cemetery (Credit: MHARP).

During analysis, the cranial morphology of the individual was noted as being of an unusual type and unlike other individuals from the cemetery (Fig 4). Therefore, the cranial measurements (S1 Table) were inputted into FORDISC and CRANID, with additional measurements being taken where necessary. The individual was found not to have an affinity with any of the populations contained within the program databases, which do include some from northern Europe, although not Britain. Therefore, the individual could be said not to share a physical affinity with these northern European samples, although this should not be taken as implying anything about their specific identity or origin. Populations that are poorly represented in the database include those from southern Europe and northern Africa (with the exception of Egypt), so there is a possibility that the individual could share physical cranial affinities with such populations, as his cranial morphology does bear similarities to other individuals from British archaeological populations who were also unclassifiable by FORDISC and have been suggested, on isotopic data, to originate from these areas [20]; (Stephany Leach personal communication, 2012).

#### Controls

Sk1 –This individual was an old middle adult male. There was evidence for a number of common pathological conditions and *ante-mortem* trauma but no evidence for any skeletal changes associated with leprosy.

Sk12 –This individual was an old middle adult male. There was evidence for a number of common pathological conditions and *ante-mortem* trauma but no evidence for any skeletal changes associated with leprosy.

### Biomolecular findings

#### Screening for *M*. *leprae* DNA

**Fig 5:**
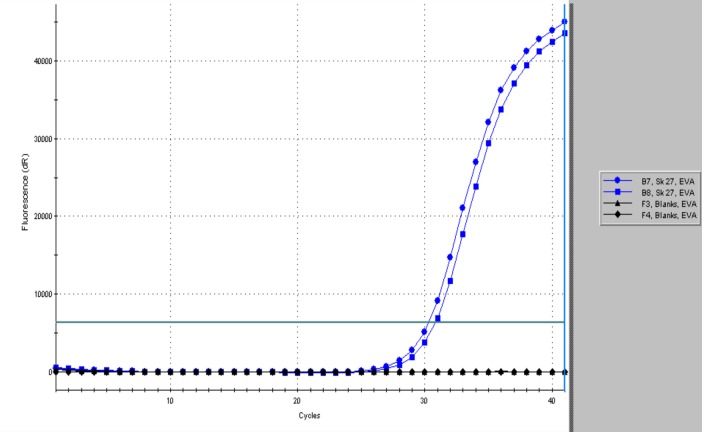
RLEP RT-PCR Amplification profiles of Sk27 and controls Sk1 and Sk12, showing formation of 111 bp product monitored with EVAGreen

**Fig 6:**
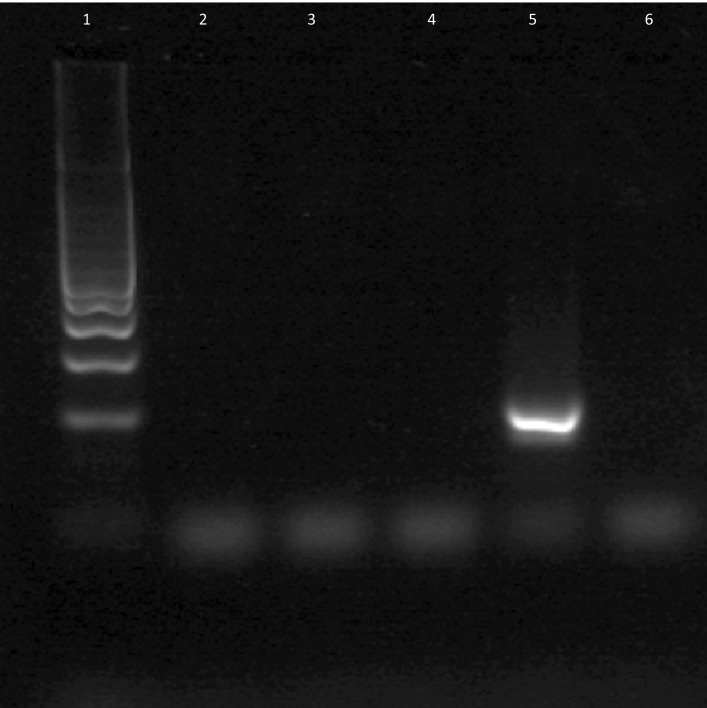
Gel electrophoresis of 111 bp RLEP PCR product run out on 3% agarose. Lane 1, 100 bp DNA size markers. Lane 2, Sk1 (palate). Lane 3, Sk1 (nasal conchae). Lane 4, Sk12 (nasal conchae). Lane 5, Sk27 product (nasal conchae). Lane 6, template blank.

**Fig 7:**
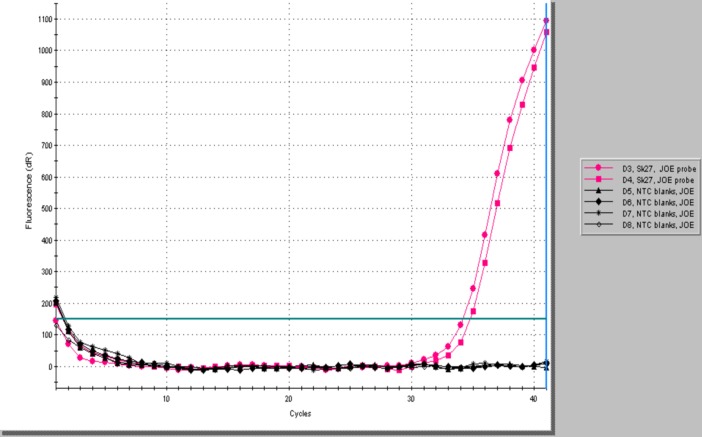
18-kDa RT-PCR Amplification profile of Sk27 showing formation of product monitored with a specific dual-labelled hybridisation probe.

The pilgrim burial Sk27 was found to be strongly positive for the RLEP repetitive sequence even in skeletal elements not displaying signs of leprosy, with Cq values of 30 and below (Fig 5 and S1 Fig). Melt curve analysis of the RLEP products showed a single product with dissociation of the strands in the expected temperature range of 91-94C, much higher than for primer-dimer or other non-specific products (S2 Fig). Agarose gel electrophoresis confirmed formation of a single band of the expected size (111 bp, Fig 6). Confirmation for the presence of *M*.*leprae* DNA in this case was provided by the 18-kDa real time PCR, with product (114 bp) reported using a specific dual-labeled probe (Fig 7). The two other individuals Sk1 and Sk12, lacking any osteological signs of leprosy, were found to be PCR negative using both leprosy DNA loci. We view this as a significant finding as both control cases have previously proved positive for human DNA [4].

Table 3 summarizes the aDNA tests performed on Sk27 and the burials selected as controls. Note that the Cq values for the RLEP assay of Sk27 were consistently lower than for the 18-kDa PCR, reflecting the higher copy number of the former (n = 37) present in the *M*. *leprae* genome. All template and extraction blanks were negative, indicating that cross-contamination was not an issue in the *M*. *leprae* tests.

**Table 3:** Summary of real-time screening PCR data for the pilgrim burial Sk27 and control cases Sk1 and Sk12. RLEP and 18-kDa PCR methods for *M*.*leprae* and IS*1081* for *M*.*tuberculosis* complex.

Burial	Sex	Age (yrs)	Sample extract	RLEP PCR	18-kDa PCR	IS*1081* PCR
				(Cq ^a^)	(Cq ^a^)	(Cq)
Sk27	Male	18–25	1.Nasal conchae	++	+	-
Pilgrim				(27.84)	(34.53)	(No Cq)
			2.Skull fragment	++	+	ND
				(27.81)	(33.62)	
			3.Foot bone	+	±	ND
				(32.58)	(39.89)	
			4.Rib	++	±	ND
				(30.54)	(38.40)	
Sk1	Male	36–45	1.Palate	-	-	-
Control			2.Nasal conchae	-	-	-
Sk12	Male	36–45	Nasal conchae	-	-	-
Control						

+ = PCR positive. - = PCR negative.

Cq = cycle of quantification

^a^ = mean of duplicates.

ND = not determined.

#### Leprosy genotyping

SNP typing. The first series of loci to be amplified and sequenced were those described by Monot and colleagues [58], namely nucleotide position 14,676 (SNP1), 1,642,879 (SNP2) and 2,935,693 (SNP3). These convey information on the main SNP type. In the case of Sk27, these were found to be C, T and A respectively indicating a SNP-type 2 (Table 4). Further sub-typing was undertaken using other phylogenetically informative loci [25]. The most relevant of these for type 2 strains were 1,104,235 and 3,102,787. Both loci were found to be C, indicating subtype F [59]. The remaining SNPs and Indel_17915 (Table 4) are less useful for genotyping type 2 strains, providing more information on type 3 isolates, particularly 3I, which is the other predominant strain to be found in burials from the Winchester leprosy hospital site [4] and reported elsewhere in medieval Britain [22]. However, the polymorphisms they demonstrate in Sk27 are entirely consistent with a SNP type 2F isolate.

**Table 4:** Sk27- summary of genotyping data.

SNP position in TN^1^ genome.	Sk27 result.	Inference.
14,676	C	
1,642,879	T	
2,935,693	A	Type 2
1,133,495	T	2E-2H
7,614	C	
1,113,926	A	
1,104,235	C	2E or 2F
3,102,787	C	2F-2H
Indel_17915 11bp repeat	2 copies	Not 3I
**Overall**		**Type 2F**
VNTR loci.		
AGA(20), ML2345	12 copies	
GTA(9), ML2172-3	7 copies	
21–3, ML0058	2 copies	

^1^TN = Tamil Nadu strain of *M*. *leprae* with nucleotide positions updated by Monot et al, 2009 [26].

#### VNTR typing

Additional VNTR typing of the 2F leprosy strain infecting individual Sk27 showed a 12-7-2 fingerprint for loci (AGA)20, (GTA)9 and 21–3 respectively. Whilst we have previously demonstrated two other cases with type 2F strains of *M*.*leprae* from the Winchester *leprosarium*, Sk14 and Sk8, both of these exhibited a 14-8-2 fingerprint [4]. Thus, the strain recovered from Sk27 is genetically distinct from 2F isolates previously studied at this site.

#### Screening for other pathogen DNA

All three individuals tested negative for MTB complex DNA using the IS*1081* repetitive element (Table 3). We used primers that amplify a 79 bp fragment of this locus, so that even extremely degraded DNA would have been detected. Bone extracts from Sk27 were also screened for known pathogenic species of *Brucella*, *Burkholderia*, *Treponema*, *Plasmodium* and *Leishmania* genera in an attempt to shed light on possible co-infections We also tested the burial for hepatitis B virus DNA, often reported to be a complication in extant cases of LL. All tests for these pathogens were negative (Table 1).

#### Radiocarbon dating

The Pilgrim burial Sk27 was radiocarbon dated to between 1020–1162 (cal AD, 95.4% probability, SUERC-39676) [6].

### Carbon and nitrogen isotope data for Sk27

#### Sample preservation

All samples from the Hospital of St Mary Magdalen yielded adequate collagen for carbon and nitrogen stable isotopic analysis in triplicate, with collagen yields in the range of 3.01% to 16.11%. All collagen values, including those for Sk27, produced atomic C/N ratios that fall within the 2.9–3.4 range, indicative of good preservation [60,61].

**Fig 8:**
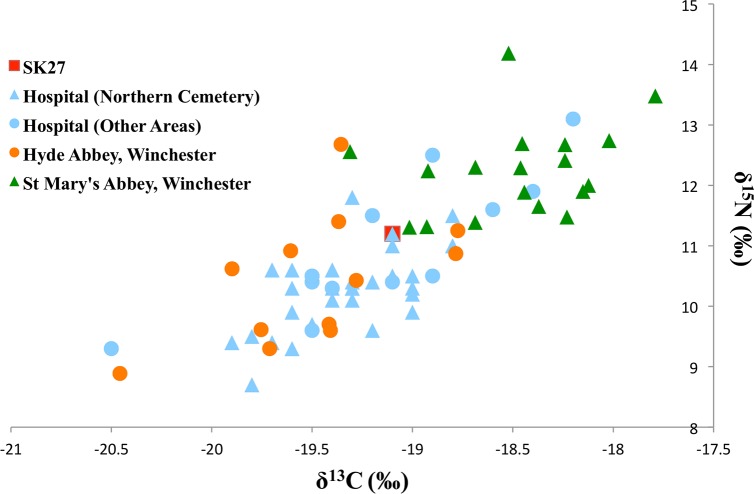
Rib collagen δ^13^C and δ^15^N results from Sk27 and nearby medieval populations.

The results for individual Sk27 are discussed in relation to the overall figures for the site, and compared with readings obtained from nearby medieval sites. (Table 5 and Fig 8). The rib sample from Sk27 produced a δ^13^C value of -19.0‰ and a δ^15^N value of 11.2‰. These values lie towards the more enriched end of the group of individuals sampled from the northern cemetery of the Hospital of St Mary Magdalen (n = 31), in use from the eleventh to mid-twelfth century [2] for both carbon and nitrogen. The values for Sk27 are also more enriched than the majority of the total hospital population (n = 43) sampled, with respect to both carbon and nitrogen. The relatively high δ^15^N value suggests that this individual consumed a diet richer in animal protein than the majority of the group, possibly complemented by marine protein, which might account for the level of ^13^C enrichment also seen. The higher carbon and nitrogen isotopic values seen in this individual compared to others from the northern cemetery, and across the site as a whole, may suggest a richer diet, potentially supporting the suggestion [62] that this was an individual of high status, or that they were a recent incomer to the population.

**Table 5:** The mean and range of δ^13^C and δ^15^N values obtained from rib collagen from Sk27, the wider cemetery population of the Hospital of St Mary Magdalen and the nearby abbey sites of St Mary’s and Hyde. These figures include values obtained from one hospital individual (Sk8), [4].

Group	N	Mean δ^13^C	Range δ^13^C	Mean δ^15^N	Range δ^15^N
Sk27	1	-19.0‰	-	11.2‰	-
Hospital of St Mary Magdalen (N. Cemetery)	31	-19.3‰	-19.9‰ to -18.8‰	10.3‰	8.7‰ to 11.8‰
Hospital of St Mary Magdalen (Total)	43	-19.3‰	-20.5‰ to -18.2‰	10.5‰	8.7‰ to 13.1‰
Hyde Abbey, Winchester	12	-19.5‰	-20.5‰ to -18.8‰	10.4‰	8.9‰ to 12.7‰
St Mary’s Abbey, Winchester	18	-18.5‰	-19.3‰ to -17.8‰	12.3‰	11.3‰ to 14.2‰

Comparison with isotopic data obtained from remains at nearby medieval monastic sites in Winchester, at Hyde Abbey and St Mary's Abbey (Nunnaminster), indicates that the values of Sk27 are more similar to the carbon and nitrogen isotopic values of individuals from Hyde Abbey, than those from St Mary’s (Table 5). Sk27 has lower nitrogen isotopic values than the individuals sampled from St Mary’s Abbey (n = 18), although the carbon isotopic value falls within the range of this group. Compared to the individuals from the site of Hyde Abbey (n = 12), Sk27 has carbon and nitrogen isotopic values close to the centre of the range of values in both carbon and nitrogen, though higher than the mean carbon and nitrogen isotopic values in both cases.

The nunnery at St Mary’s Abbey may represent an elite community, originally a royal foundation, further attested to by the high prevalence of monolithic stone coffins located within the body of the Abbey church [63]. The Abbey was situated in the south-eastern quadrant of the city close to the site of the Cathedral, royal palace and the Bishop's palace at Wolvesey. The Abbey at Hyde was founded in the northern suburb of the city and although it may not have had, traditionally, a status comparative with St Mary's, it was associated with the reburial of the Saxon King Alfred whose body was translated there in the early 12^th^ century. The elevation of St Mary's population in both carbon and nitrogen isotopic values is likely due to high consumption of marine protein. This is supported by faunal data from the site, which comprised some twenty species of fish, as well as documentary records of the purchase of fish by the abbey in the fifteenth century and the presence of a fishpond in the sixteenth [64]. The isotopic results for each site may represent contrasting dietary requirements, or regulations, between a community of men and a community of women. Overall, the comparative status of Hyde Abbey would have been significantly higher than the general urban populace overall and Sk27 appears to be similar to the Hyde Abbey population. Although the elevation of carbon and nitrogen isotopic values in Sk27 compared to the rest of the hospital group suggest an input of marine protein, this was likely not as substantial as that consumed by individuals buried at St Mary’s Abbey.

#### Strontium and oxygen

The strontium and oxygen isotope ratios and strontium concentration (Table 6) of SK27’s tooth enamel are consistent with a childhood spent in Britain, but the strontium isotope composition of 0.7103 is higher than that expected from someone whose childhood was spent in an area underlain by the chalk upon which Winchester lies [65,66]. It is, however, a common value found widely across the sedimentary silicate rocks of southern and central England [67]. The oxygen isotope value of ^18^O_SMOW(p)_ 17.4 ‰ +/-0.3 (1sd) is close to the mean value of ^18^O_SMOW(p) =_ 17.7 ‰ for British archaeological tooth enamel [68]. When converted to precipitation this gives a value of 7.2‰ +/- 0.4 (1sd) [31] which is within the range of precipitation values in Britain but is low enough to suggest this individual did not spend their childhood in the more extreme western coastal areas of Britain. The combined strontium and oxygen isotope ratios of this individual’s third molar is not unique to Britain and such a combination of values could also be found elsewhere in northern Europe, however the data provide no evidence for a non-British childhood, although it was probably not spent in Winchester or the surrounding area.

**Table 6:** Strontium and Oxygen Isotopic data for Sk27. **δ**^**18**^**O**_**p**_ **and δ**^**18**^**O**_**dw**_ were calculated used the equations in Chenery [31] and Daux [32].

Tooth	^87^Sr/^86^Sr	Sr ppm	δ^13^C _PDB_	δ^18^O _SMOW_	δ^18^O_p_	δ^18^O_dw_
				_measured_	_calculated_	_calculated_
R. Man. M3	0.71038	72.9	-12.79	26.1	17.2	-7.2

## Discussion

### Background

Leprosy has afflicted humankind since antiquity and references to leprosy can be found in a diverse range of early sources including the Buddhist Pali Canon (first few centuries AD), early medical texts, and the Bible, as well as evidence from the archaeological record. The earliest known human remains showing suspected skeletal evidence of the disease might date back almost 4000 years BP [69]. In Britain, cases are known from the 4^th^ century AD onwards [70,71] but the disease reached endemic levels throughout the Middle Ages. This was followed by a decline from the 14^th^ century onwards, the reasons for which have been the subject of speculation [72]. Reasons put forward to explain the decline have included susceptibility of sufferers to other infectious diseases like plague, due to *Yersinia pestis*, which ravaged the population of Europe in the middle of the century, and tuberculosis, another mycobacterium against which leprosy victims would have had little resistance.

Indeed, leprosy is thought only to infect those with a predisposing genetic susceptibility. A number of human genetic factors probably influence the susceptibility to disease and its severity. These take the form of mutations in components of the innate and adaptive immune system (see refs. [73,74] for reviews of candidate genes). The subsequent clinical course is determined by the ability of the host to mount an effective cellular immune response (CMI). In tuberculoid, or paucibacillary, leprosy there are low numbers of bacilli, a good CMI response with an effective type 1-cytokine pattern and few lesions. At the other end of the spectrum, LL is characterized by numerous mycobacteria, often with disseminated sites of infection. This gives rise to the typical skeletal patterning often identified in human remains. In this form of leprosy, the T cells that mediate type 2-cytokine responses usually predominate.

In recent years, some knowledge of the nature of medieval strains of leprosy has emerged from biomolecular studies of cases from the archaeological record [25, 26,75], including whole genome retrieval of a handful of European cases [5,6]. From these studies it has become apparent that the *Mycobacterium leprae* genome has not altered significantly in any way which might explain a decline in pathogenicity since the disease was at its peak in Europe.

#### Osteology and biomolecular findings

Against this background we have examined the remains of an 11^th^-12^th^ century Pilgrim burial from the St Mary Magdalen *leprosarium* in Winchester, UK. The individual was found to be a young adult male, with very slight bony changes due to leprosy, although he may also have suffered from facial paralysis as a result of the nerve damage associated with the disease. The individual also displayed evidence consistent with *ante-mortem* dental trauma, as well as degenerative and entheseal changes that may suggest heavy physical activity, possibly associated with his Pilgrim status. The cranial morphology and metrics suggested an unusual appearance that showed no affinity with northern European samples but that may share physical characteristics with populations in southern Europe or northern Africa.

At the outset, there was little expectation that Sk27 would be a good candidate for aDNA analysis, given the minimal observable skeletal lesions. However, we examined the burial for *M*. *leprae* to assist with diagnosis, given the good DNA survival at this site [4]. The findings of a multibacillary form of leprosy were unexpected. Indeed, sampling of multiple skeletal elements showed widespread distribution of pathogen DNA and implied a greater burden in the skull than at sites of pathology in the feet (see S1 Fig). We surmise that in life this individual may have had obvious skin and soft tissue lesions which left very minor traces on the bones, but which marked him out as a victim of this disfiguring disease. This would explain why he was interred amongst fellow sufferers in the cemetery of St Mary Magdalen, Winchester.

We undertook strain genotyping. The strain genotype is unlikely to have any impact on the disease severity or progression, but it is relevant to the likely origins of the strain in geographical context, based on what is known of the spread of leprosy in antiquity [26,58]. Earlier genotyping of LL cases from St Mary Magdalen has shown that the strains of leprosy fall within two main phylogenetic lineages. The majority are genotype 3I, having homology with extant strains of this lineage. Specifically, they display polymorphisms and a deletion consistent with 3I-1 strains and as such are believed to be ancestral to the isolates of 3I-1 and 3I-2 implicated in zoonotic transmission of leprosy reported in the southern states of America such as Texas, Louisiana and Mexico [59]. We have previously identified a case from Ipswich with a similar genotype and of later date (13^th^-16^th^ century) indicating that this genotype was present in Britain for a number of centuries [22]. This is consistent with its eventual transfer to the New World by European colonists and settlers and where it can be demonstrated to this day [59].

The other lineage to be found at this site are the type 2 strains, specifically 2F and the pilgrim burial Sk27 was found to be infected with this strain of leprosy. Present day type 2 strains are normally associated with central Asia and the Middle East, but have been found in European archaeological contexts from Scandinavia [76] as well as from two other individuals, Sk8 and Sk14 from St. Mary Magdalen [4]. The presence of two strain types at Winchester may reflect separate introductions of the disease into southern Britain by movement of settlers in the past. The occurrence of a type 2F strain in this individual would also be consistent with someone widely travelled or of possible foreign origin.

Given the possibility of macrophage impairment in LL and good DNA preservation at the Winchester site, we also looked for evidence of a number of other pathogens in this individual (Table 1). One often mentioned with regard to leprosy is tuberculosis [77]. However, we could find no evidence for any *Mycobacterium tuberculosis* (MTB) complex species. In some parts of the world, institutionalized patients suffering with multibacillary leprosy seem to be more susceptible to hepatitis due to hepatitis B virus [78]. Such associations have been reported in Brasil [79], Africa [80,81], India [82] and Greece [83]. We therefore also tested several extracts for evidence of HBV DNA using a sensitive PCR but this, and tests for all the listed pathogens, proved negative. We have included this data on the grounds that, in aDNA analysis, it is often recommended, but rarely followed, that negative findings should be reported. We are therefore unable to shed any light on the early death of the Pilgrim other than the confirmation of the presence of *M*.*leprae* DNA.

We did not test for *M*.*lepromatosis*, a recently identified and related mycobacterium which is associated with diffuse lepromatous leprosy (Lucio’s phenomenon) [84]. *M*.*lepromatosis* was originally reported in patients from western Mexico and the Caribbean, but increasingly of wider distribution. As mixed infections have been reported, future aDNA studies could test for *M*.*lepromatosis*, even in European contexts and novel PCR methods will be needed for this. We can be confident that methods reported here would not identify *M*.*lepromatosis* under the stringency of conditions used and this assertion is borne out by unambiguous sequencing of multiple *M*.*leprae* PCR products from Sk27.

#### Isotopic analyses

The carbon and nitrogen isotope results from the collagen of individual Sk27 suggest consumption of a diet rich in animal protein, perhaps with some marine input. Sk27’s high values compared to those of others from this cemetery indicate that this individual did likely enjoy a rich diet, lending support to the assertion that this was an individual of some means [62], or that they were a recent incomer to the population. The strontium and oxygen isotope ratios and the strontium concentration are, like the dietary isotopes, relatively common values for individuals in southern and central England although the strontium isotope ratio is too high to be consistent with origins on marine carbonate terrains such as chalks and limestones or basalts and the oxygen isotope ratio too low to indicate origins in the west of Britain. Although the data cannot rule out origins outside Britain, there are many places elsewhere in Europe which could provide a similar set of values, the most parsimonious explanation for the data is that during the formation of the third molar crown, between the approximate ages of 8 and 15 years [85], SK27 was living in southeastern or central England but not in Winchester or elsewhere on the South Downs as these are underlain by chalk.

### The results in historical context

The Sk27 burial at the *leprosarium* of St Mary Magdalen, Winchester, represents the only example of a pilgrim burial with a scallop shell in a medieval leprosy hospital cemetery. Its presence in this context is of particular interest since a possible link between the increased popularity of pilgrimage and the rise of leprosy in western Europe, during the late 11^th^ and early 12^th^ century, has been previously noted [2].

The scallop shell has been associated with pilgrimage to the shrine of St. James the Great at the Cathedral of Santiago de Compostela, Galicia, Spain, since at least 1130 [86]. Pilgrimage to the shrine at Compostela grew particularly popular in the late 11th and early 12th century with the waning of Muslim attacks on the Iberian peninsula [87]. The Santiago pilgrimage, together with Jerusalem and Rome, represented one of the three great pilgrimages of the medieval period [88] and Santiago was the only place permitted to distribute scallop shells under pain of excommunication, although “fake” shells were also thought to have been sold during the medieval period [89]. The scallop shell buried with the Winchester individual has been identified as a specimen of *Pecten maximus*, which is found in Atlantic waters, including along the Galacian coast [7, 90, 91]. It is therefore the correct species of shell that would be expected to have been given to a pilgrim who had indeed completed the pilgrimage to Santiago. Burials with pierced scallop shells are generally rare, but there are examples both from Britain [92–96], France [97, 98], Scandinavia [99], Germany [100], and a number from cemeteries along the pilgrimage route in Spain [101,102].

Throughout the medieval period the act of pilgrimage was viewed as a particularly efficacious spiritual and devotional practice and many of the medieval saints’ shrines were associated with miraculous cures and healing, including leprosy. For example, in the mid-12th century, Reginald of Durham related the story of a nobleman in southern England who conducted an experiment to determine which of England’s main cults would be most likely to cure him of the disease [103]. In England, although the principal shrines were Canterbury and Walsingham, Winchester was an important and popular pilgrim centre in its own right. Winchester in the early 12th century was a bustling and cosmopolitan city. Replete with shrines, religious institutions and hospitals, it also represented a central place in the pilgrimage landscape. Similarly, at Santiago de Compostela, during the 11th to 12th centuries, it is estimated that between 0.5 and 2 million people visited every year [104]. This could amount to over 5000 pilgrims a day.

If, as the evidence presented above suggests, the pilgrim was not local to Winchester, his presence in southern England would not necessarily have been too unusual. Winchester housed several important relics and was also central to a network of pilgrim routes in the south of England stretching from Glastonbury in the west to Canterbury in the east. To the north of Winchester lay Reading Abbey, which was one of the most important pilgrimage sites in western Europe [86] and had acquired an important relic, the hand of St James, in the 1120s [87]. Such an acquisition would have been a major draw for pilgrims. Moreover, Winchester was only 15 km from the bustling port of Southampton where many pilgrims would have arrived from, or embarked upon, pilgrimages overseas. Thus Winchester, served by its own important shrines, was a key focal point in a wider pilgrim network.

Our “Pilgrim” individual was buried in the Winchester *leprosarium* at some point in the early part of the 12^th^ Century. His burial, in an anthroporphic grave cut was associated with a building, possibly an original chapel. The grave may have been left in situ when a later chapel was constructed above it, whereas others graves had been emptied out. These observations suggested he might have commanded a degree of status in death.

The current study has shed a degree of light on the strain of leprosy from which he suffered and the skeletal signs of other problems which he endured, both related and unrelated to leprosy. The evidence found indicates that while he suffered from early-stage skeletal changes of leprosy, *M*.*leprae* DNA was recovered from diverse and macrosopically uninvolved bone samples, likely indicating that the disease was already more disseminated, possibly with soft tissue manifestations. Isotopic analysis showed he consumed a diet rich in animal protein but that he might not have been local to the chalk lands of Southern Britain. However, a number of aspects of his short life remain unknown. We cannot be sure of where he spent his early life. We do not know if he was already resident in the *leprosarium* before his pilgrimage, or whether he contracted the disease abroad and returned to Britain to end his life at St Mary Magdalen, Winchester.

### Conclusions

We have examined an 11^th^-12^th^ century “Pilgrim” inhumation from the St Mary Magdalen Hill archaeological project, Winchester, UK. The anthropomorphic grave-cut contained the remains of a young adult male, between 18–25 years old at the time of his death. The grave also contained a pierced scallop shell, the symbol of the Camino de Santiago. Although interred in the North cemetery of the *leprosarium*, there were only minimal signs of leprosy on the skeleton and these were confined to the distal ends of the pedal phalanges and the lower legs. However, aDNA analysis revealed *Mycobacterium leprae* DNA in diverse skeletal elements, suggesting this individual suffered from lepromatous leprosy and would probably have displayed soft-tissue lesions in life. Genotyping showed he was infected with a type 2F isolate of *M*. *leprae*, nowadays associated with cases of leprosy from South-Central and Western Asia [4]. Several aspects of the burial and dietary isotope analysis indicated this may have been an individual of some prestige and means, who may have been a recent incomer to the hospital population. Strontium and oxygen isotopic analyses confirmed he was not local to the Winchester region but were not able to pinpoint his precise origins. However, despite limitations when using applied analysis to infer the origin, an unusual cranial morphology pointed to possible physical affinities with populations in North Africa or southern Europe. The occurrence of a type 2F strain in this individual would also be consistent with someone widely travelled or of possible foreign origin. Further ancient genome analysis linked to population genetics can potentially provide important additional information on the genetic origin, but overall these findings confirm the benefits of a multidisciplinary approach which allows investigation of the wider relationship between leprosy, medieval pilgrimage and *M*.*leprae* transmission.

## Supporting Information

S1 Fig RLEP amplification profiles from Sk27, showing data obtained from extracts of foot bone (blue trace), a rib (green trace) and a fragment of bone from the skull (red trace). All extracts were prepared from the same weight of bone sample (50mg) and the same volume tested in duplicate.
(TIF)

S2 Fig RLEP dissociation curve profile from Sk27 nasal region (blue trace) and the two control cases, Sk1 and Sk12. This is the dissociation data from the experiment shown in Fig 5. Note the single melt peak from the nasal sample at 91C, which is the expected value for this 111 bp amplicon.
(TIF)

S3 Fig RLEP dissociation curve profiles from the experiment shown in S1 Fig showing samples of the feet (blue trace), skull (red trace) and rib (green trace) taken from Sk27. Note all exhibit peak values at 91C, expected value for RLEP amplicon.
(TIF)

S1 Table Cranial measurements of Sk27 (the definitions are taken from Wright (2012) [14]. (TIF)
