# Supplementary figures and images for: Investigation of a Medieval Pilgrim Burial Excavated from the Leprosarium of St Mary Magdalen Winchester, UK
Source: PLoS Negl Trop Dis. 2017 Jan 26;11(1):e0005186. doi: 10.1371/journal.pntd.0005186 (PMC5268360; doi:10.1371/journal.pntd.0005186)

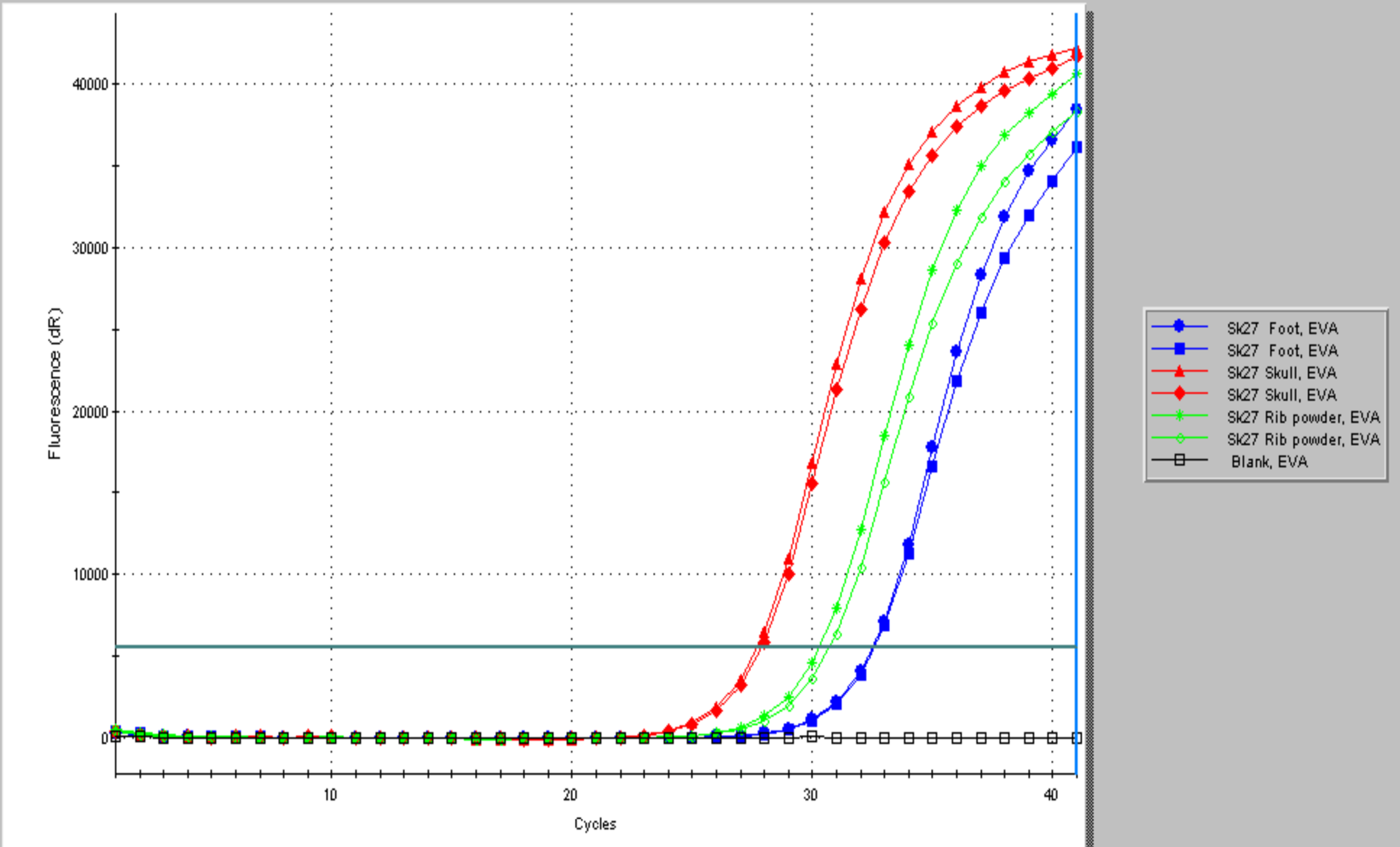

Supplement: S1 Fig — All extracts were prepared from the same weight of bone sample (50mg) and the same volume tested in duplicate. (TIF) [file pntd.0005186.s001.tif]

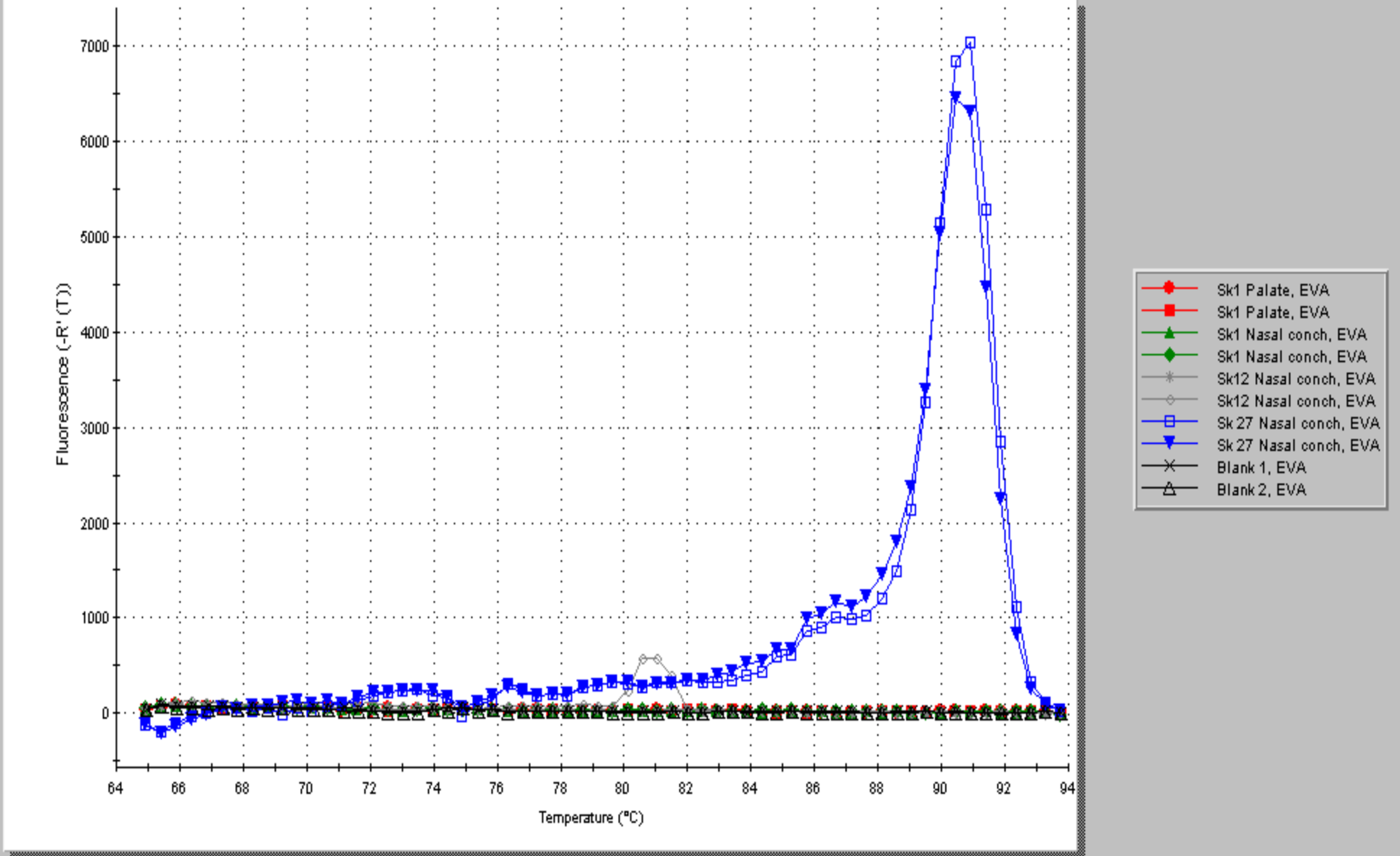

Supplement: S2 Fig — This is the dissociation data from the experiment shown in Fig 5. Note the single melt peak from the nasal sample at 91C, which is the expected value for this 111 bp amplicon. (TIF) [file pntd.0005186.s002.tif]

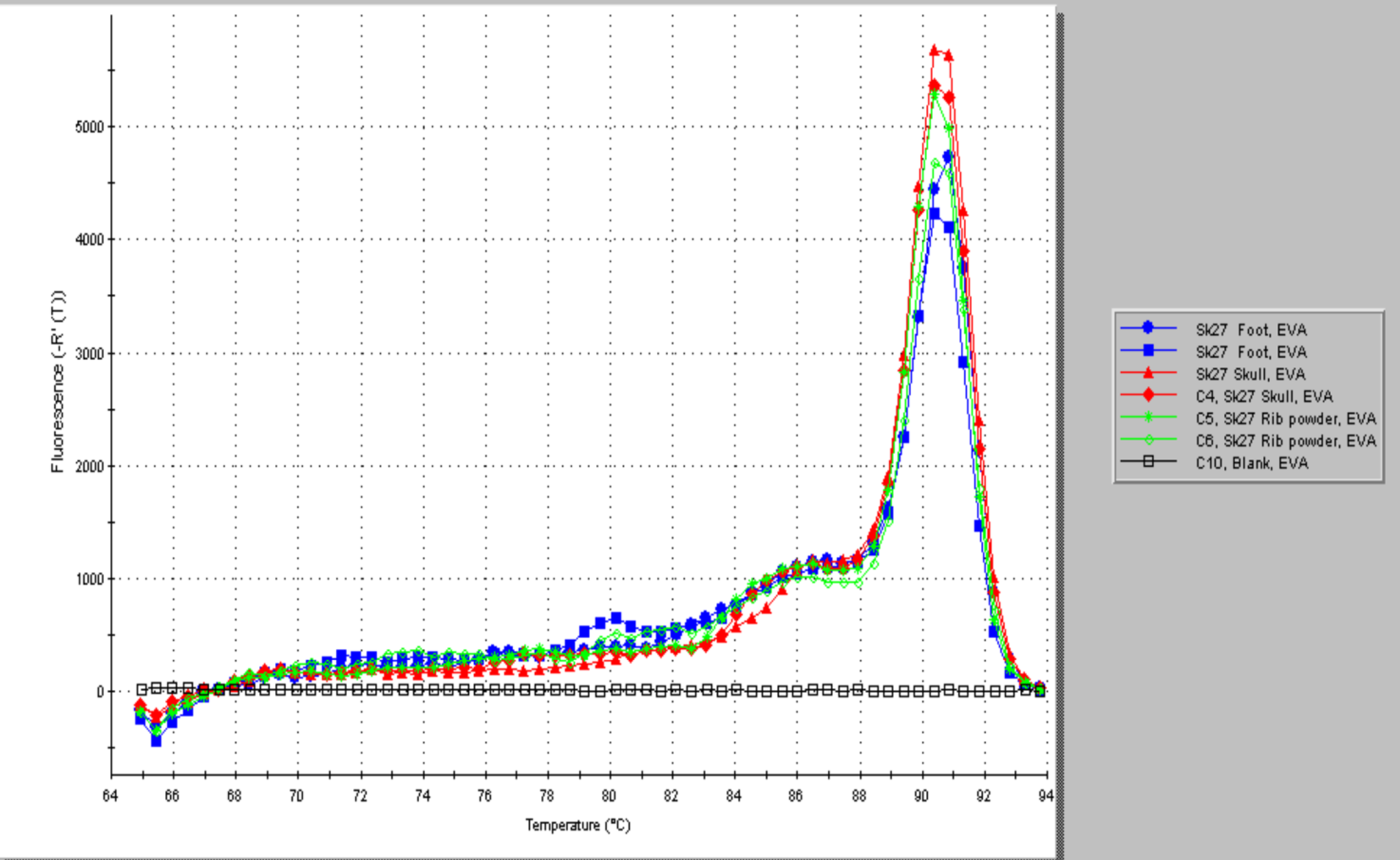

Supplement: S3 Fig — Note all exhibit peak values at 91C, expected value for RLEP amplicon. (TIF) [file pntd.0005186.s003.tif]

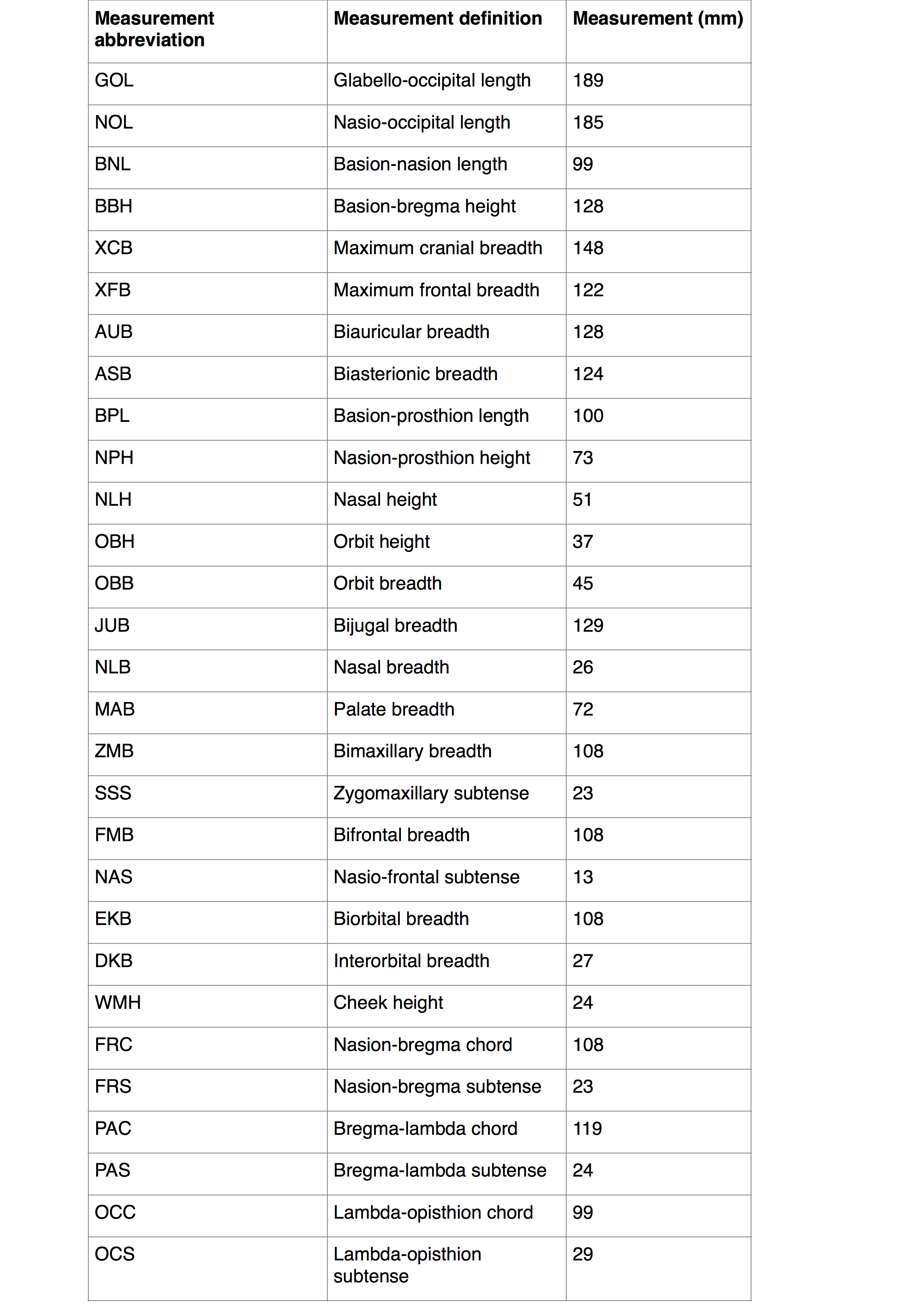

Supplement: S1 Table — (TIF) [file pntd.0005186.s004.tif]
